# Pattern Recognition Receptors and the Innate Immune Response to Viral Infection

**DOI:** 10.3390/v3060920

**Published:** 2011-06-23

**Authors:** Mikayla R. Thompson, John J. Kaminski, Evelyn A. Kurt-Jones, Katherine A. Fitzgerald

**Affiliations:** Division of Infectious Diseases and Immunology, Department of Medicine, University of Massachusetts Medical School, Worcester, MA 01605, USA; E-Mails: MikaylaR.Thompson@umassmed.edu (M.R.T.); John.Kaminski@umassmed.edu (J.J.K.); Evelyn.Kurt-Jones@umassmed.edu (E.A.K.-J.)

**Keywords:** pattern recognition receptor, toll like receptor, nod like receptor, AIM2 like receptor, RIG-I like receptor, cytosolic DNA sensor, inflammasome, interferon, virus

## Abstract

The innate immune response to viral pathogens is critical in order to mobilize protective immunity. Cells of the innate immune system detect viral infection largely through germline-encoded pattern recognition receptors (PRRs) present either on the cell surface or within distinct intracellular compartments. These include the Toll-like receptors (TLRs), the retinoic acid-inducble gene I-like receptors (RLRs), the nucleotide oligomerization domain-like receptors (NLRs, also called NACHT, LRR and PYD domain proteins) and cytosolic DNA sensors. While in certain cases viral proteins are the trigger of these receptors, the predominant viral activators are nucleic acids. The presence of viral sensing PRRs in multiple cellular compartments allows innate cells to recognize and quickly respond to a broad range of viruses, which replicate in different cellular compartments. Here, we review the role of PRRs and associated signaling pathways in detecting viral pathogens in order to evoke production of interferons and cytokines. By highlighting recent progress in these areas, we hope to convey a greater understanding of how viruses activate PRR signaling and how this interaction shapes the anti-viral immune response.

## 1. Introduction

Cells of the innate immune system utilize pattern recognition receptors (PRRs) to identify viral pathogens by engaging pathogen-associated molecular patterns (PAMPs). Once thought to be moieties found only on pathogens our understanding of PAMPs (pathogen associated molecular patterns) has expanded to include not only classical PAMPS such as lipopolysaccharides found on bacteria but also nucleic acids. Nucleic acid sensing has emerged as a major component of the immune systems anti-microbial arsenal. A diverse range of pathogens are sensed via recognition of their genomes or nucleic acids which accumulate during their replication. Nowhere is this more prevalent than in viral detection. PRRs respond to signatures present in viruses such as 5′ triphosphate RNA, which is not normally found in host RNA or to nucleic acids such as viral DNA which is exposed to sensors localized in the cytoplasm.

Of the PRRs, the Toll-like receptors (TLRs) are perhaps the most extensively studied. TLRs are type 1 transmembrane proteins that traffic between the plasma membrane and endosomal vesicles. They are primarily responsible for detecting PAMPs in the extracellular environment. Those located on the plasma membrane are usually specific for hydrophobic lipids and proteins while those found in endosomes detect nucleic acids. This segregation appears intentional allowing innate cells to respond to components of the viral envelope such as fusion machinery at their surface. In contrast, nucleic acids are detected in the endosome where many viruses uncoat their genomes and enter the cytoplasm. Upon reaching the cytoplasm, viral components are subject to the scrutiny of the retinoic acid-inducble gene I-like receptors (RLRs), the nucleotide oligomerization domain-like receptors (NLRs) and cytosolic DNA sensors such as members of the AIM2 family. Similar to TLRs, RLRs and DNA sensors regulate transcription factors essential for the production of interferons and cytokines. In contrast, NLRs and AIM2 are mainly responsible for the maturation of IL-1β and IL-18 through the activation of caspase-1. Interestingly, the immature forms of IL-1β and IL-18 are induced by TLR signaling while NLRs act as a ‘checkpoint,’ regulating the activation and release of these potent effectors. In addition to the production of proinflammatory molecules, many classes of PRRs mobilize the adaptive immune response by increasing expression of MHC class II and inducing expression of the costimulatory molecules CD40, CD80 and CD86.

## 2. The Toll-like Receptors

The Toll protein was first recognized for its role in dorsal-ventral patterning of *Drosophila* embryos. Later studies found it to be important for the adult fly’s immune response to bacterial and fungal infections fueling the search for mammalian homologs. To date, 10 TLRs have been identified in humans, 13 in mice with TLRs 1–9 common to both. TLR1, TLR2, TLR4, TLR5 and TLR6 are located on the plasma membrane while TLR3, TLR7, TLR8, and TLR9 are endosomal. All TLRs share a common architecture consisting of extracellular leucine-rich repeats and a cytoplasmic Toll/Interleukin-1 Receptor (TIR) domain [1]. These receptors signal as dimers, differentially recruiting the adaptor proteins Mal (MyD88 adapter-like), also called TIRAP (TIR domain-containing adaptor protein) and MyD88 (Myeloid differentiation primary response gene 88) and/or TRIF (TIR-domain-containing adaptor inducing IFNβ) and TRAM (Trif-related adaptor molecule) [1]. Adaptors initiate signal cascades culminating in the activation of nuclear factor kappa b (NF-κB), mitogen-activated protein kinase (MAPK) and interferon regulatory factors 1, 3, 5 and 7 (IRF-3, -5 and -7) [2]. Together these transcription factors not only drive expression of interferons, cytokines and chemokines but also influence cellular maturation and survival.

### 2.1. TLR Signaling

With the exception of TLR3 all TLRs recruit MyD88 upon activation. In the case of TLR2 and TLR4, the Mal/TIRAP protein acts as a bridging adapter to recruit MyD88 to the activated receptor [3]. MyD88’s death domain associates with and activates IL-1R-associated kinase 1 (IRAK-1) and/or IRAK-2. IRAK-4 also transiently interacts with this complex and is thought to phosphorylate IRAK-1. IRAK-1 is subsequently released and engages TNFα receptor-associated factor 6 (TRAF6). Activated TRAF6 is capable of K63-linked polyubiquitination of itself and other proteins. It interacts with NF-κB essential modulator (NEMO, also known as IKKγ), another of its ubiquitination targets, as well as TGF-β-activated kinase-1 (TAK1) and the TAK1 binding proteins (TAB1, TAB2 and TAB3). NEMO forms a complex with IKKα and IKKβ, which are the catalytic kinases responsible for phosphorylating IκB. IκB binds to and sequesters NF-κB in the cytoplasm. Following phosphorylation, IκB is ubiquitinated and finally degraded by the proteasome releasing NF-κB to enter the nucleus and induce gene expression. Studies indicate that TAK1 plays an essential role in both the NF-κB and MAPK pathways by phosphorylating IKKβ and c-Jun N-terminal kinase (JNK), respectively [4,5].

TLR3 is incapable of recruiting MyD88 and instead interacts with the adaptor protein TIR-domain-containing adapter-inducing interferon-β (TRIF). TRIF can directly bind TRAF6 and induce NF-κB in a manner similar to MyD88. In contrast to MyD88, TRIF is also able to recruit the protein receptor interacting protein-1 (RIP-1). RIP-1 synergizes with TRAF6 resulting in more potent NF-κB activation. A third protein recruited to TRIF is TRAF3. TRAF3 associates with TANK binding kinase-1 (TBK1) and IKKi and is essential for the production of type I interferon. TBK1 and IKKi mediate this production by phosphorylating interferon regulatory factor-3 (IRF3) and IRF7. This allows them to dimerize and enter the nucleus where they cooperate with NF-κB and activator protein 1 (AP-1) to bring about target gene transcription. TLR4 can recruit TRIF through the adaptor TRIF-related adaptor molecule (TRAM) and can therefore signal through either pathway.

A number of primary immunodeficiencies in humans are the result of defects in the innate signal pathways described above. For instance, one study of children with nonfunctional MyD88 proteins found they were predisposed to recurrent life-threatening pyogenic bacterial infections [6]. A similar phenotype has been reported in patients with IRAK-4 deficiency [7]. A study of two unrelated children with defects in UNC-93B1, a protein thought to be involved in trafficking TLR3, TLR7, TLR8 and TLR9 to the endosome, found an increased susceptibility to encephalitic herpes simplex virus-1 infection [8]. PBMCs and fibroblasts derived from these children demonstrated a reduced type I interferon response to HSV-1 challenge and a concomitant enhancement in viral replication [8].

### 2.2. TLR Expression and Activity

The inflammatory response evoked by viral PAMPs depends on a variety of factors. Firstly, cellular expression of TLRs differs between innate cell types. Human macrophages are known to express high levels of TLR2 and TLR4 while plasmacytoid dendritic cells (pDCs) mainly express TLR7 and TLR9 [1]. Expression patterns also vary between species, where TLR9 is restricted to a few cell types in humans it is widely distributed in mice. Furthermore, expression of certain downstream signaling molecules fluctuates between innate cell types. For example, pDCs are unique in that they constitutively express the transcription factor IRF7 allowing them to quickly produce high levels of type I IFNs in response to viral infection while other cell types such as macrophages may respond in a more delayed manner [2,3]. Thus, the response to identical viral PAMPs may differ between cell types both in the nature of effector molecules produced and the kinetics of the response. Virally encoded proteins that subvert or distort the TLR response often further complicate this picture. In the subsequent sections we discuss the TLRs individually, detailing the viruses they detect and wherever possible the specific viral products sensed.

### 2.3. TLR4

The TLR4-mediated response to LPS is well known for its critical role in innate immune control of Gram-negative bacterial infection. It was also the first TLR shown to respond to a viral pathogen. In 2000, Kurt-Jones *et al.* reported the interaction between the fusion (F) protein of respiratory syncytial virus (RSV) and TLR4 [4]. The importance of TLR4 in human viral disease and RSV pathogenesis has been documented in genetic studies. In humans, inheritances of two different single nucleotide polymorphisms (SNPs) in the ectodomain of TLR4 are associated with reduced responses to both LPS and RSV F. A highly significant association was found between RSV infection in high-risk infants and inheritance of hyporesponsive TLR4 SNPs [5]. This was confirmed in a separate study that likewise found a significant association between these same TLR4 SNPs and severity of RSV disease in infants [6].

Initial studies linking TLR4 expression to RSV pathogenesis were done in the TLR4-deficient mouse strain C57BL10ScNCr (which has a deletion of the gene region containing TLR4) as well as in C3H/HeJ mice (non-signaling point mutation of TLR4) [4,7]. These studies found that RSV activated NF-κB in a TLR4-dependent manner at early time points of infection [8]. The original RSV infection studies with ScNCr mice were controversial as it was suggested that the failure to control RSV was due to a defect in IL-12R signaling [9]. However, this discrepancy between the different studies was due in part to confusion about the mouse nomenclature since the ScNCr mice used in the initial studies (but misidentified as ScCR in the paper [4]) have normal IL-12R [10] while the ScCr mice used by the second group were IL-12R-deficient [9]. More recent work using targeted TLR4 knockouts on a B6 background (with normal IL-12R) have confirmed the role of TLR4 in controlling RSV replication independent of IL-12R, but interestingly these studies have also revealed an even more important role for TLR2 in limiting RSV replication [11]. The purified F protein of RSV induced IL-6 production in a dose-dependent manner in human peripheral blood mononuclear cells (PBMCs) and wild type mouse macrophages alike. However, this response was lost in TLR4 deficient and TLR4 knockout macrophages [4,11]. Studies by Vogel and colleagues have shown that the ability of TLR4 to be triggered by RSV F is critical to prevent RSV-induced pathology. Indeed, the formalin-inactivated RSV vaccine which caused exacerbated disease in clinical trials and was found to contain a denatured, non-stimulatory F protein. The disease enhancing activity of the formalin-inactivated RSV vaccine could be reversed by the addition of MPL, a non-toxic lipid A TLR4 agonist [12]. Disease severity is also correlated with the absence of “alternatively activated” (AA) macrophages that play a crucial role in tissue repair [13]. Taken together with the human and mouse genetics, these studies suggest that TLR4-F protein interactions may protect the host from severe RSV disease by mitigating or reprogramming the host response to promote AA-macrophages and thus promote healing [14].

TLR4 is also important for infections by the retrovirus mouse mammary tumor virus (MMTV). MMTV was shown to activate NF-κB and induce B220 and CD69 lymphocyte activation markers in B cells from wild type but not C3H/HeJ or congenic BALB/c (C.C3H Tlr4^lps-d^) lines [15]. TLR4 activation, attributed to the envelope (Env) protein, was found to stimulate production of IL-10 [16]. Surprisingly induction of TLR4 signaling appears to benefit MMTV. First, it activates quiescent B cells encouraging cell division, which is necessary for viral genome integration in the host chromosome. Secondly, it promotes secretion of IL-10, an immunosuppressive cytokine that helps the virus persist indefinitely [15].

### 2.4. TLR2

Functional TLR2 exists as a heterodimer with either TLR1 or TLR6 on the plasma membrane of both innate and adaptive immune cells. It can be activated by lipoteichoic acid, a common component of gram-positive bacteria, as well as GPI anchors of parasitic protozoan such as *Plasmodium falciparum*. The TLR2/TLR6 heterodimer has recently been shown to play a role in the innate immune response to RSV. Macrophages from mice deficient in TLR2 or TLR6 responded to RSV with lower levels of TNFα, IL-6, CCL2 (MCP-1) and CCL5 (RANTES) than their wild type counterparts. When TLR2 or TLR6 knockout mice were challenged intranasally with RSV they had elevated peak viral titers and lower numbers of neutrophils and activated DC in their lungs [11]. Thus, TLR2/TLR6 signaling likely contributes to both innate immune cell recruitment and viral clearance *in vivo* during RSV infection [11]. In human PBMCs, TLR2 contributes to IL-8 and MCP-1 production in response to Epstein-Barr virus (EBV) [17]. A TLR2/TLR1-mediated proinflammatory response to the related human cytomegalovirus (HCMV) has also been reported. One study found TLR2 deficient mouse macrophages had significantly reduced IL-6 and IL-8 production in response to UV-inactivated HCMV [18]. Furthermore, expression of TLR2 and CD14 was required for maximal NF-κB activation and IL-8 secretion in HEK293 cells exposed to HCMV. Envelope glycoproteins B and H were later shown to coimmunoprecipitate with TLR2 and TLR1 and are theorized to be the HCMV PAMPs stimulating TLR2 [19].

Lymphocytic Choriomeningitis (LCMV) is a non-cytolytic virus that can cause fatal encephalitis in mice. Wild type glial cells infected with LCMV produce TNFα, CCL2 and CCL5, a response that is abolished in cells derived from TLR2 deficient mice [20]. TLR2 also induces MHC class-I and class-II, CD40 and CD86 expression in microglia challenged with LCMV, implicating this pathway in the induction of adaptive immunity [20]. In LCMV infection, where much of the CNS damage is caused by the immune response itself, it remains to be determined if TLR2 signaling is protective or pathological. Interestingly, TLR2 is important for type I IFN induction during LCMV infection but the mechanism is unclear [21]. Although TLR2 is normally not associated with type I IFN induction, a recent study from Barton and colleagues demonstrated that on inflammatory monocytes, TLR2 regulates induction of type I interferon in response to viral but not bacterial ligands [22].

Surprisingly, it appears TLR2 can play either a protective or detrimental role in disease caused by herpes simplex virus (HSV) depending on the context of the infection. Studies using an intraperitoneal infection model found TLR2 deficient neonates were protected from lethal HSV-1 encephalitis compared to wild type mice [23]. Despite having similar viral loads, the TLR2 knockouts demonstrated improved survival, attenuated symptoms and reduced CNS inflammatory lesions. In contrast, TLR2 was shown to work synergistically with TLR9 to promote survival in an intranasal HSV-1 infection model [24]. In addition, TLR2 has been shown to be beneficial in both intraperitoneal and intravaginal HSV-2 infection models [25]. TLR2’s role in murine HSV infection models may be influenced by factors such as the size of the viral inoculum, the route of administration and the age of the subject. HSV induced two distinct responses; a TLR2-dependent inflammatory cytokine response and a TLR9 and/or non-TLR-dependent type I IFN response. A strong IFN response is necessary to control early virus replication (IFN-deficient mice quickly succumb to infection) and prevent spread from the genital tract to the brain [25]. Once in the brain, however, inflammation is linked to increased mortality [23].

Measles virus (MV) is another infection in which TLR2 signaling may have both favorable and unfavorable effects. Challenging mice with live or UV-inactivated wild type MV induces IL-6 production and CD150 surface expression in mouse macrophages; a response that is impaired in TLR2-deficient cells [26]. Intriguingly, CD150 is required for entry of wild type MV into monocytes, thus immune activation through TLR2 may in fact benefit the virus by conferring susceptibility. This study identified MV hemaglutinin (HA) protein as the viral PAMP triggering TLR2 activation [26]. MV vaccine strains carrying a single asparagine to tyrosine substitution in the HA protein lacked the ability to activate TLR2.

### 2.5. TLR3

With the exception of neutrophils and pDCs, TLR3 is widely expressed in innate immune cells where it is localized to the endosomal compartment [27,28]. In 2001, Alexopoulou *et al.* demonstrated that activation of TLR3 signaling by the double stranded RNA analog poly(I:C) contributed to the production of type I IFN and cytokines in macrophages. Moreover, genomic dsRNA isolated from reovirus was found to activate wild type but not TLR3 deficient splenocytes. The idea that TLR3 could respond to dsRNA, a common viral PAMP, led to intense speculation about its role in the host response to numerous infections. Counterintuitively, a later study found no difference in the survival, viral titers or pathology of TLR3 deficient mice following reovirus challenge [29]. The authors suggested that during *in vivo* infection, TLR3 may not encounter reovirus dsRNA or that levels may be too low to efficiently activate TLR3 [29]. This study also reported indistinguishable immune responses to LCMV, VSV and MCMV infection in TLR3 deficient and wild type mice [29]. However, other evidence exists suggesting that TLR3 does in fact play a role in controlling MCMV as some studies observed blunted type I IFN and IL-12 production accompanied by higher viral loads in the spleens of mice lacking TLR3 [30,31]. Despite this, only TLR9 deficient mice had significantly decreased survival compared to wild type suggesting TLR9 is more crucial than TLR3 in MCMV infections [30]. A recent study also implicates TLR3 in immune suppression of the related herpes virus HSV-1. Patients with TLR3 dominant negative mutations were found to be more susceptible to herpes simplex encephalitis, a rare but devastating manifestation of HSV-1 infection [32]. The presumed ligand for TLR3 in infections with DNA viruses is dsRNA generated during bidirectional transcription of opposing DNA strands. TLR3 signaling also reduces lethality of encephalomyocarditis virus (EMCV), a ssRNA virus that directly damages heart tissue [33]. TLR3 deficient mice challenged with EMCV had decreased levels of TNFα, IL-6 and IL-1β mRNA in cardiac tissue and a corresponding reduction in inflammatory infiltrate at 3 days post infection [33]. Without TLR3 signaling, EMCV replicated to higher levels in the heart resulting in more rapid and extensive mortality in knockouts [33].

Although this study indicates that the TLR3-mediated inflammatory response is beneficial in EMCV infections; TLR3 signaling appears to be detrimental in a number of other viral infections. For instance, TLR3 deficient mice were protected compared to their wild type counterparts when challenged with a lethal dose of West Nile Virus (WNV) [34]. This study found that TLR3 driven production of inflammatory cytokines compromised the blood-brain barrier facilitating WNV entry. This resulted in higher viral loads in the CNS and worsened neuropathology. Likewise, TLR3 was shown to play a pathologic role in infections with Punta Toro Virus (PTV) [35]. Wild type mice had drastically reduced survival and increased hepatic injury compared to TLR3 deficient mice following PTV challenge. Despite having similar serum and hepatic viral loads, wild type mice had elevated levels of IL-6, IFNγ, CCL2 and CCL5, suggesting these proinflammatory molecules may mediate much of the damage observed [35]. Interestingly, although TLR3 signaling increases inflammation and reduces Influenza A virus (IAV) lung titers, it causes a paradoxical decrease in survival. Thus, in IAV infections, lethality appears to be more dependent on TLR3 signaling than direct virus-induced injury.

### 2.6. TLR7 and TLR8

TLR7 and TLR8 are two closely related receptors that, like TLR3, act in the endosome. Human TLR7 and TLR8 were first shown to respond to the imidazoquinoline-like compound resiquimod (R-848), a synthetic drug recognized for its antiviral and antitumor activity [36,37]. We now know that nearly any long single-stranded RNA (ssRNA) is capable of activating TLR7 and TLR8 [38]. Despite this, differences do exist between these receptors. For example, short dsRNAs containing certain motifs preferentially activate TLR7 [39,40]. Furthermore, synthetic agonists specific to TLR7 or TLR8 differentially activate innate immune cells leading to distinct cytokine profiles [41]. In 2004, Diebold *et al.* showed that TLR7 mediates IFNα production by pDCs in response to live or heat-inactivated influenza virus [42]. This TLR7 response could be elicited simply by exposure to purified genomic ssRNA and was completely abrogated by chloroquine, an inhibitor of endolysosomal acidification [42]. Thus, the authors proposed a model, now known as the exogenous pathway, whereby pDCs endocytose and degrade a portion of incoming influenza virions, allowing TLR7 to engage exposed genomic RNA. A similar TLR7-dependent type I interferon response was observed when pDCs were challenged with vesicular stomatitis virus (VSV) [43]. Under normal circumstances both influenza and VSV require endocytosis for viral entry. However, using a recombinant strain of VSV (VSV-RSV-F), capable of fusing to the plasma membrane, Lund *et al.* demonstrated that VSV activated TLR7 regardless of the route of viral entry. TLR7 is also responsible for pDC production of IFNα in response to Sendai virus (SV); another ssRNA virus which enters at the plasma membrane [44]. Interestingly studies of SV using human U937 and murine RAW 264.7 myeloid lines found only a partial role for TLR signaling in cytokine and chemokine production [45]. Recent evidence suggests the cytosolic RLR receptors are chiefly responsible for the cytokine and interferon response to SV in myeloid cell types other than pDCs [46].

One important observation gleaned from studies using SV and VSV was that, in contrast to influenza, UV-inactivation of these virions abolished TLR7 activation [44]. From this work a second model of TLR7 activation known as the endogenous pathway was proposed. According to this theory, ssRNA intermediates produced during SV and VSV infection are transferred from the cytoplasm to the endosome by means of autophagy [44]. Thus, to elicit a TLR7 response by this route, cells must be exposed to live, replication competent virus. This model is supported by studies showing that selective inhibitors of autophagy and mice deficient in autophagic pathways lack a TLR7 mediated response to SV and VSV [44]. Recent studies have implicated TLR7 and TLR8 in the response to human immunodeficiency virus (HIV). ssRNA derived from the HIV genome caused murine pDCs and macrophages and human PBMCs to produce IFNα, IL-6 and TNFα [47]. In mice this activity was TLR7-dependent while in humans it appears to rely on TLR8 suggesting that HIV receptors may be species-specific. A study by Wang *et al.* found IFNα production by human and mouse pDCs responding to Coxsackievirus B (CVB) was also dependent on TLR7 [48]. Interestingly, this response required the presence of CVB-specific antibodies as well as functional Fc Receptor complexes on the pDC surface. Thus they proposed a mechanism whereby opsonized CBV is delivered to the endosome via FcR and once internalized viral RNA is detected by TLR7 [48]. This observation suggests previous exposure to CVB can influence subsequent innate responses furthering our understanding of the complex interplay between adaptive and innate immunity.

### 2.7. TLR9

**Figure 1.**
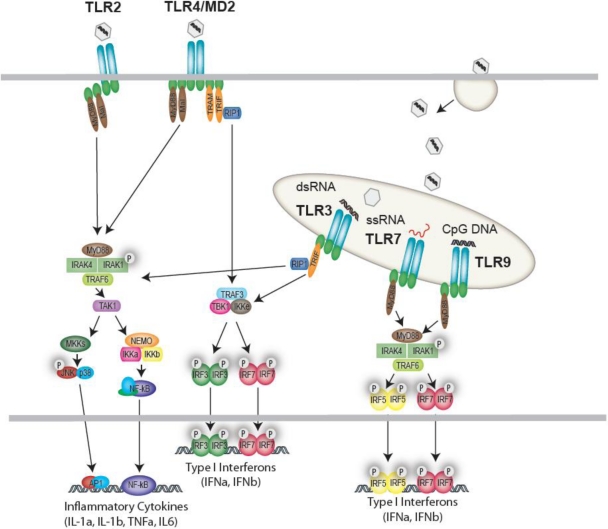
Cell surface and endosomal recognition of viruses by Toll-like receptors (TLRs). TLR2 responds to a variety of viruses resulting in activation of a MyD88-dependent NF-κB and MAPK pathway. TLR4, responding to viral proteins (e.g., RSV F-protein) activates both a MyD88-dependent and MyD88-independent response. The MyD88-dependent response leads to transcriptional regulation of inflammatory cytokines, while the MyD88-independent response is regulated via TRAM/TRIF and the IKK-related kinases which drive IRF3 activation and type I Interferon production. In the endosome, TLR3, TLR7, TLR8 and TLR9 sense viral nucleic acids and generate either IRF3 activation (TLR3) or IRF7-driven type I IFNs (TLR7, 8 and 9).

In both humans and mice, TLR9 is highly expressed in pDCs, innate cells renowned for their ability to rapidly produce large amounts of type I interferon [1]. TLR9 responds to the unmethylated deoxycytidylate-phosphate-deoxyguanylate (CpG) motifs in viral and bacterial DNA [49]. Not surprisingly TLR9 has been shown to play a crucial role in infections caused by a number of DNA viruses. For instance, TLR9 deficient mice infected with MCMV have a drastically increased mortality compared to their wild type counterparts. This hypersensitivity is likely due to the blunted type I IFN and IL-12 response and reduced NK cell activation which results in an elevated MCMV load [30]. In EBV infection, production of type I IFN, IL-6 and IL-8 by pDCs is largely dependent on TLR9 [17]. This is in contrast to monocytes where TLR2 synergizes with TLR9 to orchestrate the cytokine response to EBV [17]. TLR9 signaling also plays a role in the interferon response to HSV types I and II. One study found IFNα production by mouse pDCs in response to HSV-2 was completely dependent on TLR9 and independent of viral replication [50]. Using cholorquine it was shown that this recognition required endosomal maturation and could be evoked simply by exposure to purified HSV-2 DNA [50]. Furthermore, following *in vivo* HSV-2 challenge, IFNα was only detectable in the serum of mice with intact TLR9. A similar role for TLR9 was described in the response to HSV-1 by splenic pDCs. However, this study also described a delayed IFNα response by conventional dendritic cells (cDCs) and macrophages that was both TLR9 and MyD88-independent but required exposure to replication competent virus. The TLR9-independent IFN response is likely due to cytoplasmic RLRs and may explain why one study using TLR9 deficient mice identified no *in vivo* defects in the HSV-1 control [51]. Alternatively, TLR9 signaling may be more important in certain manifestations of HSV-1 induced disease. A recent study showed TLR9 deficient mice did have higher rates of mortality and viral replication when challenged intranasally with HSV-1 [24]. Thus TLR9’s precise role in HSV pathogenesis and the relative contributions of other PRRs requires further investigation. Figure 1 illustrates the TLRs activated by viral pathogens and depicts their downstream signal pathways.

## 3. Intracellular Nucleic Acid Sensors

As discussed above, the TLRs play an important role in sensing viral PAMPS that are present within the extracellular compartment, as well as in endosomes. In certain contexts, TLRs can receive viral nucleic acids generated from viruses that replicate in the cytoplasm, via an autophagy mechanism. A role for intracellular sensors in the clearance of viruses that replicate and reside within the cytosol of cells has recently emerged. Following the generation of mice lacking TLRs and examination of their susceptibility to virus infections, it became clear that additional sensing mechanisms must also exist and contribute to anti-viral defenses. The last decade or more has revealed numerous additional classes of innate sensors. Of particular relevance to anti-viral defenses was the discovery of specialized classes of cytosolic nucleic acid sensors, termed RIG-I like receptors (RLRs), which recognize intracellular RNA that is introduced to the cytosol during viral infection or that accumulates during replication. Additionally, a diverse selection of intracellular DNA sensors which recognize viral DNA within the cytosol have also emerged.

### 3.1. The RIG-I like Receptor Family

The RLR family is comprised of three DExD/H box RNA helicases: retinoic acid-inducible gene (RIG-I), melanoma differentiation-associated gene 5 (MDA-5), and laboratory of genetics and physiology-2 (LGP-2) [60–64]. Both RIG-I and MDA-5 are comprised of tandem N-terminal caspase activation and recruitment domains (CARDs) followed by a DExD/H box RNA helicase domain which has ATPase activity and a C-terminal repressor domain (RD). Unlike RIG-I and MDA-5, LGP-2 lacks the N-terminal CARD domains, containing only the RNA helicase domain. As such, LGP-2 was postulated to act as a negative regulator of the other RLRs [61,63]. Under resting conditions, RIG-I resides in the cytoplasm in an inactive form that is auto inhibited by its regulatory domain. Upon viral infection, RIG-I undergoes a conformational change by which it dimerizes in an ATP dependent manner [63]. The activated multimeric form of RIG-I or MDA5 then interacts with the downstream adaptor protein mitochondrial antiviral signaling protein (MAVS), also known as VISA, IPS-1, and CARDIF, via CARD-CARD interactions. MAVS is localized to the outer leaflet of the mitochondrial membrane, which is an essential location to support downstream signaling. Recently, MAVS was also shown to be localized on peroxisomes, from where it induces an early antiviral response through the direct induction of a subset of anti-viral genes via the transcription factor IRF1. Upon engagement of RIG-I or MDA5 with MAVS, MAVS activates the IKK-related kinase, TBK1/IKKi, which activates IRF3/IRF7, resulting in the transcription of type I interferons. MAVS also activates NF-κB through recruitment of TRADD, FADD, caspase-8, and caspase-10 [65–69].

### 3.2. RNA Recognition by RLRs

The RLRs are critical components of the anti-viral defense pathway in many cell types including fibroblasts, epithelial cells, and conventional dendritic cells [70]. Initially, it was thought that both RIG-I and MDA-5 recognized the synthetic dsRNA, polyinosinic acid (polyI:C). However, studies from RIG-I and MDA-5 deficient mice determined that MDA-5 alone was responsible for interferon production by polyI:C stimulation [71]. Instead, RIG-I recognizes 5′-triphosphorylated, uncapped ssRNA, which is a common feature in many viral genomes. However, it is unable to recognize the capped 5′-ppp ssRNA from the host cell [72–74]. These finding suggest that RIG-I uses the 5′ end of a transcript to discriminate between viral and host RNA. MDA-5 distinguishes between viral and host RNA not by its 5′ end, but rather by the length of the RNA sequence; long dsRNA is not naturally present in host cells and acts as a ligand of MDA-5. In addition to recognizing 5′-triphosphate RNA, RIG-I is also capable of recognizing short dsRNA, which is produced as a byproduct of viral replication [75].

RIG-I and MDA-5 appear to differentially recognize different classes of RNA viruses. Studies involving RIG-I deficient mice implicated RIG-I in the recognition of vesicular stomatitis virus (VSV), rabies virus, SV, Newcastle disease virus (NDV), RSV, measles virus, Influenza A and B, hepatitis C virus (HCV), Japanese encephalitis virus, and ebola virus [53,70,71,76–78]. Studies from MDA-5 deficient mice show that MDA-5 is able to recognize EMCV, theiler’s virus, and mengo virus [71,77]. All of these viruses do not contain a 5′ triphosphate RNA, but are able to produce long dsRNA, providing further evidence that MDA5 discriminates between self and non-self RNA based on sequence length and not the 5′triphosphate. More recently studies have shown that both CVB and poliovirus are dependent on MDA-5 for type I IFN production [79,80]. Moreover, some viruses, such as dengue, West Nile virus, and reovirus, signal through a combination of both RIG-I and MDA-5 [79,81,82].

As discussed above, LGP-2 lacks N-terminal CARD domains, and was first thought to be a negative regulator of RLR function [61,63]. Initial studies found that overexpression of LGP-2 decreased the capacity of SV and NDV to induce interferon production. Evidence that LGP-2 could associate with RIG-I through mutual RD domains led to the proposal that LGP-2 directly prevented RIG-I association and activation. Consistent with this idea, interferon signaling was found to be increased in LGP-2 deficient mice responding to polyI:C, providing evidence for negative regulation of MDA-5 as well [83]. A second *in vivo* study using LGP-2 deficient mice as well as mice harboring an inactive ATPase in the DExD/H-box RNA helicase domain showed that LGP-2 acted as a positive regulator of RIG-I and MDA-5-mediated signaling after infection by RIG-I and MDA-5-specific RNA viruses. This phenotype is consistent with the possibility that LGP-2 might promote RNA accessibility, thus enabling RIG-I or MDA-5 dependent viral recognition. Further studies on these mice will no doubt clarify this upstream mechanism and the role of LGP-2 in this pathway.

### 3.3. DDX3

Another member of the DExD/H box RNA helicase family, DDX3, has also recently been implicated in anti-viral defenses. Schroder *et al.* found that the vaccinia virus protein K7 inhibited IFNβ induction by binding to DDX3, which led to the discovery that DDX3 had a positive role in the RLR signaling pathway [84]. A more recent study reported that DDX3 binds to both polyI:C and viral RNA introduced into the cytosol and associates with MAVS/IPS-1 to upregulate IFNβ production. These results led the authors to speculate that DDX3 might enhance RNA recognition, forming a complex with RIG-I and MAVS to induce interferon production [85]. Further studies are required to determine whether DDX3 is a *bona fide* RNA sensor or a component of the RLR signaling pathway in order to fully understand the function DDX3 plays in anti-viral surveillance and signaling.

### 3.4. Cytosolic DNA Sensors

Prior to the discovery of TLR9, it was known that DNA derived from pathogens could activate fibroblasts to produce type I IFNs [86]. This phenomenon was ignored or underestimated for decades and was rediscovered following the observation that transfection of pathogen-derived dsDNA activated a TLR9 negative thyroid cell line to upregulate various immunological genes [87]. Akira and colleagues subsequently demonstrated that TLR9−/− MEFs, which failed to respond to CpG DNA, produced large amounts of IFN in response to transfection with synthetic b-form dsDNA or genomic DNA isolated from bacteria, viruses, and mammalian cells [87]. This was similar to findings presented by the Medzhitov lab using a 45 bp dsDNA region from the *Listeria monocytogenes* genome. Cytosolic administration of dsDNA did not appear to utilize any known TLRs to induce interferon since cells from mice lacking both MyD88 and TRIF responded normally.

Like the cytosolic RNA recognition pathways, cytosolic DNA recognition also leads ultimately to activation of TBK1 and IRF-3 and production of type I IFNs. However, the signaling pathway linking upstream DNA sensors to TBK1 are poorly characterized. TBK1 associates with DDX3, a DEAD box RNA helicase, which regulates IFNβ transcription via IRF-3 [84,85]. In addition, TBK1 interacts with the exocyst protein Sec5 in a complex that includes the recently identified endoplasmic reticulum (ER) adaptor stimulator of interferon genes (STING) [69,88–90]. STING plays a central role in the signaling pathway upstream of TBK1 following HSV infection [69]. STING also interacts with the ER translocon components Sec61β and TrapB in a manner essential for regulation of cytosolic DNA-induced type I IFN production, although the mechanistic understanding of this finding is not known [88]. In unstimulated cells, STING localizes to the ER and perhaps ER-associated mitochondria [90]. Following stimulation with cytosolic DNA and HSV-1, STING translocates to perinuclear foci, via the Golgi [88]. STING localizes partially to endosomes, particularly Sec5 positive structures [88], whilst another report has demonstrated that STING localizes to vesicular structures, which are not peroxisomes, mitochondria, endosomes or autophagosomes [91]. Further work is required to clarify the precise subcellular localization of STING. What is clear is the essential role of STING in cytosolic DNA sensing pathways. Much less clear is the mechanisms or receptors which act upstream of STING. A growing number of DNA sensors have now been implicated and will be outlined below.

### 3.5. DAI

DNA-dependent activator of IFN-regulatory factors (DAI) was among the first of the cytosolic DNA sensors to be discovered. It is composed of two binding domains for left-handed, Z form DNA, although the protein can recognize B form DNA as well. When DAI was exogenously expressed in L929 cells, it increased type I IFN production in a dose dependent manner following stimulation by both B and Z form DNA. Similarly, knockdown of DAI with siRNA impaired type I IFN production in response to DNA, the 45 bp interferon stimulatory DNA (ISD) from *Listeria* and the herpesvirus, HSV-1 [92,93]. The production of type-1 interferons by fibroblasts in response to HCMV was also found to be dependent on DAI [94]. DAI-knockout mice were subsequently generated, and surprisingly, cells derived from DAI deficient mice respond normally to synthetic and viral dsDNA [92,95]. These results suggested that DAI might play a cell type specific, and redundant role in sensing cytoplasmic DNA, and that other sensors must also be necessary for inducing these responses.

### 3.6. RNA Pol III

As discussed above, both synthetic and viral RNA trigger the production of type I IFNs via RIG-I. Although, the RLRs are sensors of RNA, some data has suggested a role for this system in detection of DNA. A somewhat surprising finding was that synthetic B-form dsDNA can also induce IFNβ production in human cells in a manner that was dependent on the RIG-I adapter molecule MAVS [52–54]. These findings suggested the existence of an unknown DNA sensor that would signal via MAVS. Recently, two independent studies have provided an explanation for these findings and shown that AT-rich DNA can be transcribed by RNA polymerase III into 5′-ppp RNA, which subsequently activates RIG-I [52,55]. This pathway was reported to be involved in type I IFN induction during EBV infections where the EBERs are transcribed by RNA polymerase III [56]. This indirect DNA-sensing system was also reported to be involved in induction of type I IFN following HSV-1 or *Legionella* infection [52,55,57].

### 3.7. LRRFIP1

In addition to DAI and RNA Pol III, Leucine-rich repeat flightless-interacting protein 1 (LRRFIP1) has recently been implicated as a regulator of DNA-driven innate immune signaling. LRRFIP1 was found to bind to the drosophila homolog flightless I and play a role in actin organization during drosophila embrogenesis. In a study using *Listeria monocytogenes* to screen for potential cytosolic DNA sensing molecules, siRNA against LRRFIP1 was found to inhibit type I IFN production induced by the bacteria. The authors showed that the IFN response to VSV was dampened in these cells as well. Furthermore, knockdown of LRRFIP1 inhibited IFN production in response to polyI:C, and the synthetic DNA species, poly(dG:dC) and poly(dA:dT), implicating LRRFIP1 in the recognition of both dsRNA and both B and Z form dsDNA. Surprisingly, this function is independent of RNA Pol III. LRRFIP1 does not regulate IRF3 activation but instead appears to regulate a novel β-catenin-dependent coactivator pathway. LRRFIP1 binds RNA or DNA and leads to phosphorylation of β-Catenin, which subsequently translocates to the nucleus where it associates with the p300 acetyltransferase at the IFNβ1 promoter, leading to increased IFNβ production [101]. Although LRRFIP1 has been implicated in the recognition of both *Listeria monocytogenes* and VSV, further studies are needed in order to determine its role in sensing other viruses, particularly DNA viruses.

### 3.8. IFI16

While analyzing immune responses to a dsDNA region derived from the VV and HSV-1 genomes, Bowie *et al.* identified IFI16 as a DNA binding protein which interacted with these dsDNAs. IFI16 is a member of the PyHIN (pyrin and HIN200 domain-containing) protein family. The PHYIN family consists of 4 family members: IFIX, IFI16, MNDA and AIM2. All contain one or more HIN200 domains, which recognize DNA as well as a pyrin domain. Knockdown of IFI16 or p204 (a member of the murine PYHIN family) led to a reduction in IFNβ responses to these dsDNAs while responses to the RNA virus SV was unaffected. Although IFI16 is primarily nuclear in most cell types, in macrophages IFI16 also localized to the cytosolic compartment where it co-localized with dsDNA introduced via lipofectamine. Association of IFI16 with STING was required for the production of IFNβ in response to these DNA motifs. siRNA knockdown of IFI16, and its mouse homolog p204 led to a decrease in IRF3 and NF-κB activation and IFNβ gene induction following infection of cells with HSV-1 [102].

### 3.9. DDX9 and 36

Also in the family of DExD/H box RNA helicases, DHX9 and DHX36 have recently been shown to recognize and bind CpG-B and CpG-A DNA, respectively in plasmacytoid dendritic cells. Activation of DHX9 leads to IRF-7 activation and IFNα production, while activation of DHX36 leads to the activation of NF-κB and the production of IL-6 and TNFα. siRNA knockdown of DHX9 and DHX36 inhibited cytokine production in response to the DNA virus HSV-1, while response to the RNA virus influenza A was unaffected [103].

## 4. Inflammasomes

Although the sensing of cytoplasmic DNA is linked to the transcriptional induction of type I IFN and other pro-inflammatory cytokines, cytosolic DNA has also been shown to trigger the caspase-1-dependent maturation of the pro-inflammatory cytokines IL-1β and IL-18 [104,105]. IL-1β, a close biological relative of TNFα, is involved in innate cell recruitment, activation of T-lymphocytes and induction of fever [106]. IL-18 increases the cytolytic activity and IFNγ production of natural killer (NK) cells and influences neutrophil recruitment and activation [106,107]. Growing evidence supports the importance of these cytokines in anti-viral defenses [108,109]. Mice lacking either one of these cytokines have demonstrated enhanced susceptibility to influenza A virus and HSV-1 infections [110]. Moreover, pretreating mice with IL-18 protects them from subsequent HSV-1 and VV challenge [111,112].

In contrast to type I IFNs and TNFα, the production of IL-1β is controlled at the level of transcription, translation, maturation and secretion [113,114]. Many cell stimuli including TLR-ligands activate the transcription of the pro-forms of IL-1β and IL-18. Unlike most other cytokines however, these pro-cytokines lack leader sequences and are retained in the cytoplasm rather than loaded into secretory vesicles. Maturation (*i.e.*, the cleavage) of pro-IL-1β and pro-IL-18 is catalyzed by the cysteine protease caspase-1 (formerly known as IL-1 converting enzyme). In resting cells, caspase-1 itself is present as an inactive zymogen pro-caspase-1 [115]. A large ‘inflammasome protein complex’ controls the activity of the inflammatory caspase-1 [115]. Several protein complexes have been shown to form inflammasomes upon recognizing specific stimuli. NLRPs 2 to 14, which contain a C-terminal LRR-rich domain, a central nucleotide-binding NACHT oligomerization domain, and an N-terminal protein–protein interaction pyrin domain (PYD) associate with the PYD containing adaptor molecule apoptosis-associated speck-like protein (ASC; also termed pycard or TMS1) [116]. ASC links the NLRP’s via its C-terminal CARD domain to the CARD domain of pro-caspase-1. This close association of pro-caspase-1 molecules is then believed to provoke self-cleavage into active caspase-1. Active caspase-1 then cleaves pro-IL-1β and pro-IL18. ASC is critical for caspase-1 activation in response to many stimuli [106,107,115,117,118].

### 4.1. AIM2

Cytosolic dsDNA also triggers an ASC dependent activation of caspase-1 resulting in the maturation and secretion of IL-1β and IL-18. These findings suggested the existence of an inflammsome complex that can be triggered by DNA. Analysis of this response in macrophages lacking members of the NLRs revealed normal caspase-1 activation in these cells. Subsequent studies from several groups revealed that this response was instead dependent on AIM2 (Absent in melanoma-2), an interferon inducible protein that belongs to the same PYHIN family as IFI16 [105,119–121]. AIM2 recognizes cytosolic dsDNA of self and nonself origin including viral DNA via its HIN200 domain in a sequence-independent manner. Contrary to other cytosolic sensors of DNA, the recognition of DNA by AIM2 triggers the assembly of an inflammasome complex. Upon DNA binding, AIM2 likely undergoes oligomerization and associates with ASC via homotypic pyrin-pyrin domain interactions, which in turn recruits pro-caspase 1. Published data has shown that the AIM2 inflammasome is an integral component of innate sensing of DNA viruses [109]. AIM2 is essential for the activation of caspase-1 and proteolytic processing of IL-1β and IL-18 in antigen presenting cells in response to infection with MCMV and VV. Furthermore, AIM2-ASC dependent IL-18 secretion and NK-cell activation is critical in the early control MCMV infection *in vivo* [105,109]. In addition to viruses, AIM2 has also been shown to recognize *Francisella tularensis* and as observed for DNA viruses appears to be critical in early control of *Francisella tularensis* infection *in vivo*. Moreoever, AIM2 as well as NLRP3 and IPAF function in a redundant manner in the recognition of *Listeria monocytogenes* [109,122].

### 4.2. NLRP3

In addition to the AIM2 inflammasome, a number of recent studies have shown that mice deficient in NLRP3 are more susceptible to virus infections, particularly RNA viruses [104,123,124]. Loss of NLRP3 was found to attenuate the normal IL-1β and IL-18 responses to influenza virus and was associated with diminished innate cell recruitment to the lung and increased pathology [123]. Further studies revealed that influenza’s M2 protein, a proton-specific ion channel was needed to trigger the NLRP3 inflammasome [124]. Viral RNA has also been shown to trigger NLRP3 activation, although this is unlikely to be a direct RNA-NLRP3-interaction. The precise relationship between M2 and RNA in NLRP3 activation remains to be clarified. The NLRP3 inflammasome also plays a role in the response to adenovirus, a DNA virus [104]. Peritoneal macrophages isolated from NLRP3 or ASC deficient mice exposed to adenovirus are unable to secrete mature IL-1β [104]. When challenged *in vivo*, NLRP3 knockout mice had reduced levels of IL-1β, IL-6, CCL4 (MIP-1β) and CXCL10 (IP-10) in the liver. Recently, a viral NLR homolog was identified in the dsDNA virus, KSHV. The KSHV tegument protein ORF63 appears to be an NLR homolog that can inhibit inflammasome activation by binding to NLRP1 and NLRP3 [58]. Inflammasome activation suppresses KSHV reactivation from latency, suggesting that inflammasome activation and IL-1β mediated signaling facilitates KSHV latency. These observations are consistent with a model whereby the KSHV tegument ORF63 protein might bind NLRP3 and/or NLRP1 to block the detrimental effects of inflammasome activation.

**Figure 2.**
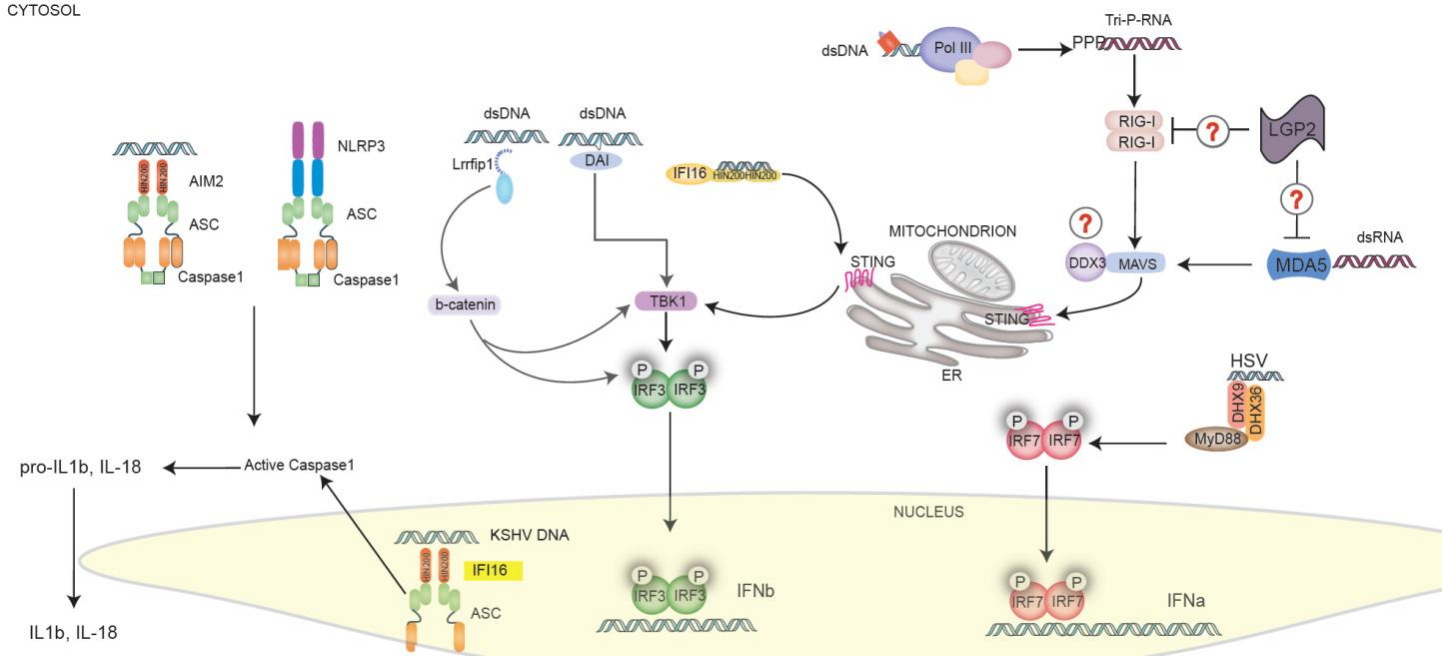
Cytosolic and Nuclear Pattern Recognition Receptors (PRRs). A multitude of DNA sensors, including IFI16, RNA Polymerase III, DAI, LRRFIP1, and DDX9/36 recognize DNA and drive type I IFNs and cytokine production. RIG-I and MDA5 recognize RNA in the cytosol. All of these molecules converge on STING in the case of DNA or MAVS in the case of RNA. STING and MAVS then engage either the TBK1-IRF3 or the IKKb-NFkB pathways, resulting in the activation of type I IFN responses and inflammatory cytokines, respectively. AIM2 (which binds to dsDNA) and NLRP3 (which can respond to viral RNA (probably indirectly)) act in the cytosol to promote the formation of a multiprotein inflammasome complex that contains the adaptor protein ASC, and caspase-1. IFI16 can also detect DNA in the nucleus during KSHV infection. Nuclear IFI16 engages ASC which then triggers caspase-1 in the cytosol. Activation of caspase-1 results in the proteolytic cleavage of pro-IL-1β and pro-IL-18 to IL-1β and IL-18, respectively. The mature cytokines can then be released from the cell.

Intriguingly, a recent study has revealed a role for IFI16 in the recognition of Kaposi sarcoma-associated herpesvirus (KSHV) in endothelial cells. IFI16 is known to recognize viral DNA in the cytosol and drive type I Interferon production, as discussed above. In endothelial cells however, IFI16 in the nucleus can sense the KSHV DNA and form a complex with the inflammasome adapter molecule ASC. These findings suggest that IFI16 can form an inflammasome complex following recognition of nuclear DNA during infection with this virus [59]. Figure 2 portrays the cytosolic and nuclear receptors known to respond to viral pathogens and their downstream signal pathways.

## 5. Conclusions and Future Perspectives

Over the past decade our understanding of how the innate immune system detects viruses and triggers antiviral responses has increased immensely. Our knowledge of what constitutes a PAMP, once limited to classical TLR activators such as LPS, has recently expanded to include nucleic acids. This has led to the discovery of a variety of cytosolic RNA and DNA receptors and their downstream signaling pathways. Although our grasp of TLR function has matured significantly over the past decade, a number of prominent questions remain regarding cytosolic and nuclear PRR signaling. First, many of the cytosolic sensors appear to play redundant roles in viral detection. Such overlapping defense strategies may have evolved in order to combat viral evasion mechanisms. Defining the function of newly identified PRRs in immune defense to viral infection is an important step in understanding their unique or ancillary contributions to pathogenesis.

Secondly, it remains unclear how some nucleic acid sensors discriminate self from non-self. Just as RIG-I recognizes the 5′ triphosphate moiety found principally on viral RNAs, a mechanism presumably exists allowing PRRs such as IFI16 to distinguish between virally derived and host DNA. Another question that must be addressed is how viral RNA and DNA is made accessible to PRRs. For instance, it is not well understood how nucleic acids are presented to cytosolic sensors in cases such as HSV infection where viral DNA is shielded by a capsid in the cytoplasm and replicates within the nucleus. As we explore these and other questions it is imperative that we apply our findings in human model systems. By encouraging cooperation between basic and clinical communities we can ensure that new discoveries are quickly translated into therapeutic strategies.
